# Problem Recognition as A Discrete Concept for Change Processes in Problematic Alcohol Use

**DOI:** 10.1007/s40429-025-00634-x

**Published:** 2025-02-19

**Authors:** J. Morris, D. K. Richards, I. P Albery

**Affiliations:** 1 Department of Psychology, School of Applied Sciences, London South Bank University, London, UK; 2 Center On Alcohol, Substance Use, And Addictions (CASAA), University of New Mexico, Albuquerque, USA

**Keywords:** Problem recognition, Alcohol use disorder, Insight, Behaviour change, Cognition, Recovery

## Abstract

**Purpose of review:**

Alcohol problem recognition reflects the extent to which a person with any level of problematic alcohol use (PAU), including hazardous alcohol use, acknowledges the associated risks/harms as potentially/actually problematic with a relative degree of objectivity. Notably, alcohol problem recognition is typically low amongst people with PAU not engaged in treatment or support. This review evaluates existing PAU problem recognition measures and related concepts such as ambivalence, readiness to change, motivation, cognitive biases and other self-evaluative appraisal processes.

**Recent findings:**

Alcohol problem recognition has been operationalised via various measures but is often conflated with other related but theoretically distinct concepts. Limited conceptual work examines the nature of problem recognition as a discrete concept and its function in relation to behaviour change outcomes and key variables.

**Summary:**

Problem recognition is proposed as an important theoretically distinct process that warrants further conceptual development and testing for advancing understanding of change processes across the PAU spectrum.

## Introduction

*Problematic alcohol use* (PAU) has been used to define any level of alcohol use at or above hazardous levels, including *alcohol use disorder* (AUD), thus reflecting a broad spectrum of risky and harmful drinking behaviours[1].^1^ Although various cognitive factors involved in PAU behaviour change have been studied [e.g., 2–4], limited research has specifically addressed *problem recognition*, namely, the extent to which the person explicitly identifies their alcohol use as ‘problematic’ in relation to any number of potential PAU indicators (e.g., risk or harm to health, functioning, social problems, impaired control etc.).

Whilst indicators of problem recognition processes are often presented as a crucial feature of ‘recovery’ from AUD in many scientific [e.g., 5] and lived experience accounts [e.g., 6], and within the first step of Alcoholics Anonymous [7], such accounts do not reflect scientifically developed concepts of problem recognition across the broad spectrum of PAU. Problem recognition has therefore been proposed as an important cognitive process to develop scientific understanding of [8], particularly with regard to its potential to increase intervention engagement and effectiveness [9–11] and to facilitate natural recovery processes (i.e., self-change) amongst PAU groups who do not meet AUD thresholds [8, 12].

This narrative review aims to examine key existing literature relating to PAU problem recognition and clarify the concept in order to advance future conceptual and empirical work. That is, we aim to briefly assess key applications of problem recognition with a focus on assessing its potential to assist future efforts in better understanding and reducing PAU. Notably, whilst a limited number of studies have used specific problem recognition measures, others have conflated problem recognition with related but conceptually distinct measures such as ambivalence, motivation, efficacy and help-seeking. A primary focus on empirical work is taken, with a brief review of qualitative insights into how discourse amongst people with PAU may relate to problem recognition processes. However, this is a selective review and therefore does not take a systematic approach to identifying relevant problem recognition measures. Key considerations are proposed for advancing understanding of the role of problem recognition in PAU behaviour change.

## Methods

### Narrative Review

#### Conceptualizing Problem Recognition in PAU

At present, problem recognition does not have a unified definition in the context of PAU behaviours and outcomes [9], but a number of measures have been designed to capture self-identification of ‘problem drinking’ (see Section. 3). Notably, these problem recognition measures are distinct from the self-identification of alcohol-related harms/consequences or a range of other related concepts (see Section. 4). As such, as the basis for this review we propose a broad working definition of PAU problem recognition as:

*‘… the extent to which a person experiencing a significant level of risk (i.e., hazardous alcohol use), problems or harms associated with their alcohol use explicitly recognizes these with a reasonably objective degree of evaluation*.’

**Fig. 1:**
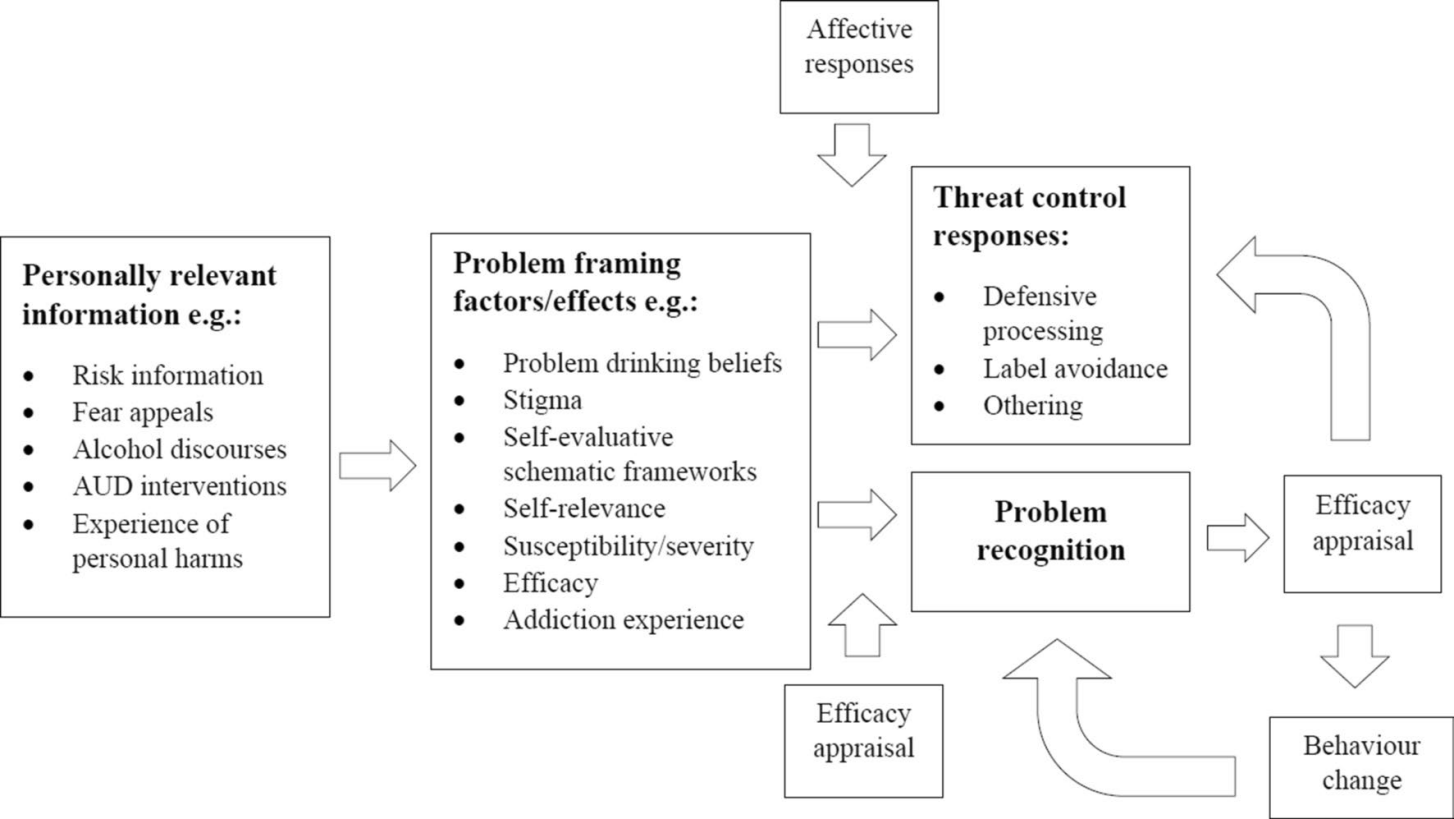
A conceptual model of problem framing factors and effects on problem recognition amongst harmful drinkers [8]

The term ‘problem’ is used to denote a wide range of potential risks (i.e., ‘hazardous’ use) or actual harms (either physical, psychological or social) associated with alcohol use [13–15]. Problem recognition is therefore a process by which recognition reflects, to a reasonable extent, objective self-appraisal of a person’s own level of risk, consequences or experienced harms associated with their alcohol use. Notably, Nye et al. [10] identified PAU problem recognition based on a self-regulation model as the discrepancy between *self-monitoring* of one’s drinking behaviour and *self-evaluation* against a perceived goal or standard. Other addiction focused work has also emphasized the role of the persons’ self-standards as integral to ‘recovery’ [e.g., 5, 16]. As such, problem recognition depends on a person’s perception of what a ‘problem’ is and is thus heavily influenced by socio-cultural [17, 18] and many other contextual cues [8, 19, 20], as proposed by a conceptual framework by Morris and colleagues (2021; see Fig. 1). It is therefore important to emphasize that problem recognition itself is subject to strong normative influences and established cognitive biases towards alcohol use [21, 22]. Low problem recognition should not therefore be considered as a ‘failure’ of the individual [23], particularly when considering normative forces and public stigma towards those perceived to be ‘problem drinkers’ [24, 25]. We therefore argue that whilst problem recognition is an important individual level process to develop understanding of, it is essential to recognize the importance of socio-cultural and environmental factors in determining drinking behaviours and reducing associated harms [26, 27].

## Problem Recognition in PAU: Direct Measures

### SOCRATES Problem Recognition Scale (SPRS)

The main direct measure of PAU problem recognition has been via the use of four items from the 19-item *Stages of Change Readiness and Treatment Eagerness Scale* (SOCRATES) [28], to our knowledge first applied by Nye et al. [10]. From the original SOCRATES, two items of the four items are derived from the Ambivalence Scale *(“There are times when I wonder if I drink too much”* and *“Sometimes I wonder if I am in control of my drinking”*) and two items from the Recognition Scale (*“If I don’t change my drinking soon, my problems are going to get worse”* and *“My drinking is causing a lot of harm”*).

In a small sample (n = 72) of US college students, Nye et al. [10] used the four item SOCRATES problem recognition scale (from here on referred to as SPRS) to experimentally test for effects of two types of alcohol brief intervention (ABI). Students exposed to a self-focusing intervention or social norms-based intervention about alcohol use had higher SPRS scores than those in a control condition. However, both alcohol interventions together were associated with lower problem recognition, which the authors posit may have resulted from heightened defensiveness when delivered simultaneously. Agostinelli et al. [29] subsequently utilised the SPRS (a = 0.85) amongst a sample of college students (n = 707). Higher consumption was associated with higher problem recognition. Normative beliefs were also associated with problem recognition such that, amongst heavier drinkers, the more they considered their drinking to be above that of other students, the greater their problem recognition. Women were reported to exhibit more accurate problem recognition, potentially reflecting greater stigma towards women’s drinking.

The SPRS was used in a two experimental studies by Morris et al. [30, 31]. In an online study recruited via social media (n = 597), harmful drinkers without addiction experience reported higher SPRS (a = 0.90) when exposed to a first person video vignette depicting a continuum model of alcohol use and problems versus a binary disease model and control [30]. In a subsequent study utilizing script based depictions, continuum beliefs were not found to be associated with higher problem recognition, but binary disease model beliefs were associated with lower SPRS (a = 0.81) when including ‘alcoholism’ terminology [31]. In an experimental study testing the role of abstinent versus drinking reduction goals in combination with continuum and binary disease model beliefs, Leonhard et al. [32] identified a subgroup of participants with PAU with higher SPRS scores (a = 0.81) when exposed to both continuum belief and moderation (non-abstinent reduction) conditions. Based on these findings, the SPRS appears to be a promising measure of PAU problem recognition and aligns with the operationalization proposed in the current review. However, further psychometric examination is warranted including extending on existing factor analysis [33] and comparison with other problem recognition measures.

### Categorical and Single Item Problem Recognition Measures

Some studies have utilized single question items to test problem recognition, including via dichotomized predictors of PAU factors such as treatment engagement. Smith et al. [9] identified seven studies utilizing binary (i.e., yes/no) approaches to PAU problem recognition such as *“Do you currently think of yourself as a problem drinker?”* [34]. In a study using a single binary item of problem recognition *(“Have you ever thought you had a drinking problem?”*), Glass et al. [11] found problem recognition mediated 97.6% of the total relationship between PAU symptoms and PAU help seeking in a sample of children of twin fathers (n = 1,073). In a study assessing differences in people in remission from AUD, Cunningham et al. [35] assessed problem recognition (*“Did you used to have a problem with alcohol but no longer do?”)*, finding current abstainers to have higher problem recognition than current moderate drinkers (but also higher past AUD severity). As a single item continuous measure, Cheers et al. [36] asked participants to *“Rate on a scale of 1–10 how concerned are you about your drinking (with 1 being not concerned at all, and 10 being very concerned)?”,* finding higher concern predictive of higher threat to drinking identity-related measures.

The Hanil Alcohol Insight Scale (HAIS) is a 20 item self-report scale attempting to capture *insight* into problem drinking via both assessing alcohol-related consequences (“I find many problems in my drinking”) and recognition (“I am an alcoholic”). Raftery et al. [37] identified seven studies using the HAIS within which most adopted a three category approach to scoring problem recognition of ‘poor’, ‘fair’ or ‘good’. On account of its use of stigmatizing terminology^2^ found to have negative effects on problem recognition [31], this scale is advised against by authors and broader addiction terminology guidance [e.g., 38], though a revised version may warrant further examination (Table 1).

**Table 1:** Summary table of PAU problem recognition concepts/measures

Concept/measure	Summary	Example items	Implications for proposed problem recognition concept and development ^a^
SOCRATES problem recognition scale (SPRS)	The SPRS is a four item scale derived from the *Stages of Change Readiness and Treatment Eagerness Scale* [28], with two sub-scales (Ambivalence, Recognition) The SPRS has been used in multiple studies [e.g., 29] assessing PAU problem recognition (see 3.1)	Ambivalence scale: • *There are times when I wonder if I drink too much”* • *Sometimes I wonder if I am in control of my drinking* Recognition Scale: • *If I don’t change my drinking soon, my problems are going to get worse* • *My drinking is causing a lot of harm*	The SPRS appears to be a valid and appropriate measure of problem recognition utilized in a number of studies The SPRS may be a suitable measure for advancing problem recognition development, but should be tested against other items
AUDIT-P	AUDIT-P consists of seven items made up of 4–10 of the full AUDIT (Alcohol Use Disorders Identification Test) [39]. These items cover a range of self-evaluated problems associated with alcohol use including dependence, cognitive and social problems	Sample items include: • *How often during the last year have you failed to do what was normally expected from you because of your drinking?* • *How often during the last year have you needed an alcoholic drink in the morning to get yourself going after a heavy drinking session?* • *Have you or somebody else been injured as a result of your drinking?*	As part of the AUDIT identification tool, AUDIT-P items are widely utilized within the context of alcohol brief interventions. However, in terms of utility for capturing problem recognition (rather than identification per se; see 4.1), AUDIT-P items warrant further exploration in comparison to other measures such as SPRS
Motivation to change	Motivation to change has been widely conceptualized according to the *transtheoretical model* (TTM), which proposes five key stages of change, with a shift from *pre-contemplation* to *contemplation* strongly reflecting the concept of problem recognition. Despite its popularity, the TTM has important limitations (see 4.2). Notably, *self-determination theory* (SDT) has been proposed as a promising approach for developing understanding of motivation to change processes. A shift from controlled (e.g., external punishments) to autonomous motivation (e.g., conscious valuing) may be indicative of problem recognition	Motivation to change has been most widely measured via the *Stages of Change Readiness and Treatment Eagerness Scale* [28]*,* with four items being used specifically for the SPRS Additional motivation to change items include: • *As of now how important is it for you to make a change in your drinking* [40]*?* SDT • *[Reducing/quitting drinking]… is an important choice that I really want to make* [41]	Motivation to change is of high conceptual importance to problem recognition and further investigation is required to understand how it is best captured and related to problem recognition processes, including via further application of SDT
Ambivalence	Ambivalence is commonly understood as perceiving reasons both for and against drinking, thus may be understood as a state in which problem recognition and/or motivation to change is hindered by such discrepancy	Typically captured via Decisional Balance Scale items that might assess pros and cons for drinking [42] • *Drinking makes me feel more relaxed* • *My drinking sometimes causes problems in my relationships with others*	Ambivalence may reflect one specific aspect/factor within problem recognition, thus further work to establish its role in problem recognition processes is required
Treatment/help-seeking intentions and engagement	Engagement with help/support or intentions to seek help/support are important in improving PAU outcomes and have sometimes been used as a proxy for problem recognition	• *During the past 12 months, did you need treatment or counseling for your alcohol or drug use* [43]*?*	Whilst treatment or support help-seeking/intentions may be a useful proxy for problem recognition they are not sufficient to capture the complexity of problem recognition processes and different routes to PAU resolution (including natural recovery)
Expectancies	Expectancies are positive or negative evaluations about drinking outcomes and are established predictors of drinking behaviors and PAU outcomes [44]	The Alcohol Expectancies Scale [45] includes items such as: • *Alcohol makes social gatherings more enjoyable* • *“Drinking makes me feel more relaxed and at ease* • *“After a few drinks, I feel more outgoing and friendly*	Expectancies are important predictors of PAU behaviour change and therefore warrant further exploration in relation to problem recognition
Normative misperception	*Normative misperception* reflects the extent to which people inaccurately assess the behaviour of others (e.g., the proportion of people who drink above guideline levels)	• *How do you think your use of alcohol compares to other people who have used that substance recently* [46]*?*	Social norms are an established predictor of drinking behaviour in certain contexts [47], thus further examination of their role in problem recognition processes is warranted
Unrealistic optimism/optimism bias	*Unrealistic optimism* (or *optimism bias*) are cognitive biases in which people underestimate their own susceptibility to negative health outcomes relative to others	Unrealistic optimism scores are typically assessed by calculating a difference score between self-appraised risk and that attributed to the average person via two items [21]: • *How likely do you think it is that you will develop a drinking-related problem at some time in your life?* • *How likely do you think it is that the average person will develop a drinking related problem at same time in their life?*	Cognitive biases are proposed as an important factor in understanding problem recognition processes [21], thus warrant further empirical testing
The Hanil Alcohol Insight Scale (HAIS)	The HAIS has been used in a number of studies but utilizes a categorical scoring system and includes stigmatizing terminology in its original form [48]	• *I find many problems in my drinking”* • *I am an alcoholic*	Given ‘alcoholic’ terminology has been associated with low problem recognition [31], the HAIS is advised against in its original form
Single item problem recognition measures	Single items that assess problem recognition have been utilized in some studies	• *“Do you currently think of yourself as a problem drinker?”* [34] • *“Have you ever thought you had a drinking problem?”* [11]	Since some single items have been utilized, they warrant further examination in comparison to other problem recognition scales
Insight (‘anosognosia’)	Insight has been used in some substance use and mental health studies to evaluate problem recognition, typically within medicalized contexts. Relatedly, anosognosia has been used to refer to a ‘lack of knowledge of a disease’ [49]	See [50] for an example of how PAU insight has been measured	Insight and anosognosia may be of use in specific medical/treatment contexts, for instance in cases where severe alcohol-related brain damage may impair insight, thus should be considered distinct from problem recognition
‘Denial’	*Denial* is a lay term commonly used in recovery narratives, or by the public, for instance, to denote low problem recognition in others	NA	‘Denial’ is advised against as a term/concept for a range of reasons, including simplistic and stigmatizing connotations (see Section. 5.)

^a^ Based on author assessment of proposed definition of problem recognition (see Section. 2.)

## Related Problem Recognition Concepts and Measures

In the following section we briefly review key concepts and measures that are of relevance to the understanding and development of PAU problem recognition processes. Our goal here is to highlight these as existing operationalizations of relevance to problem recognition (see Table 1) but may require further empirical and conceptual work to examine their validity and utility for advancing efforts to reduce PAU.

### Problem Identification: AUDIT-P

Since problem recognition includes explicit recognition of negative consequences, measures which specifically capture self-reported alcohol-related harms or consequences are of particular relevance. For example, the AUDIT-P (items 4–10 of the full AUDIT) [51] is widely used in alcohol research and practice, typically as part of the AUDIT assessment tool [39]. However, *identification* of drinking harms/consequences can be conceptually differentiated from problem recognition. For instance, one may *identify* negative consequences of one’s alcohol use but not consider them to be ‘harmful’ or signify a ‘problem’. Alternatively, one may think that such consequences are not serious or enduring, or are significantly outweighed by the benefits of drinking [52, 53]. Further, one may believe – and arguably legitimately so – that experiencing alcohol-related harms does not equate to being a ‘problem drinker’. Indeed, many PAU groups *other* problem drinkers as only those experiencing severe dependence or failing to meet their responsibilities [26, 54].

An important component of problem recognition therefore reflects not just the person’s *identification* of alcohol related consequences, but also their *recognition* of these as ‘problematic’ or ‘harmful’ to some degree (e.g., the SPRS includes personal evaluation of the consequences as ‘problematic’ via identifying drinking “too much” or as “causing a lot of harm”). As such, AUDIT-P has been used as a covariate in several problem recognition studies [30, 31]. Other studies have also identified alcohol-related consequences as separate from problem recognition factors [e.g., 39].

### Motivation to Change

According to the transtheoretical model (TTM), changes in addictive behaviours are the result of progress through five stages of change (SoC) that represent increasing readiness (or motivation) to change (RTC) [55]. Most relevant to the current review is progress from the first stage of *pre-contemplation* to the second stage of *contemplation* which involves becoming aware that one’s alcohol use (or other addictive behaviour) is ‘problematic’. Indeed, as described previously, items from the SOCRATES, which is based on the SoC/RTC framework of motivation to change, have been used to quantify problem recognition (see Section. 3.1.). Despite long-standing criticisms of the SoC/RTC on conceptual grounds [56] and measurement issues [57] that have received insufficient attention [58], SoC/RTC remains the most popular conceptualization of motivation to change in relation to alcohol use.

Consistent with the findings of research on the SPRS presented earlier, cross-sectional or baseline data assessing SoC/RTC and PAU severity show positive correlations among individuals who screen positive for hazardous alcohol use or indicate a desire to change [59–63], thus theoretically supporting higher PAU severity being related to higher problem recognition (if reflected by SoC/RTC). It stands to reason that those with more severe PAU (i.e., meeting AUD criteria) would be more likely to recognize their drinking as problematic, although those actively changing their drinking (and thus further along the SoC or higher in RTC) would theoretically be expected to report less severe PAU, and in turn lower problem recognition. Indeed, Richards et al. [62] found the strongest correlations for contemplation and PAU severity compared to pre-contemplation and action. In contrast, prospective associations indicate that greater baseline SoC/RTC is related to greater reductions in substance use following psychotherapy, as expected (for a meta-analysis, see [64]). However, it would be expected that those low in SoC/RTC would benefit more from motivationally focused interventions, yet the opposite has been found [65]. Perhaps the strongest evidence against the SoC/RTC framework of motivation for change, however, is the lack of support for increased SoC/RTC as a mechanism of motivational interventions for alcohol (for a recent meta-analysis, see [66]). A recent study found that increases in the *action* SoC mediated the effects of a motivational intervention for PAU, but this finding is somewhat tautological (i.e., those who endorse greater action in reducing their drinking report greater reductions in drinking) and provides little insight into the motivational processes underlying change [67].

Extensions of the SoC/RTC framework have also considered *importance to change*, which conceptually coheres strongly with problem recognition. In a recent study examining three dimensions of ‘motivation to change’ as a mediator of ABI outcomes, a self-rated importance item *(“As of now how important is it for you to make a change in your drinking?”*) was found to mediate drinking reductions, whilst self-appraised confidence and readiness to change items did not mediate drinking reductions [40]. The mediating effect of importance was also moderated by higher problem severity. In contrast, in a clinical population meeting AUD criteria (where problem recognition is expected to be relatively high), *confidence* was found to be the strongest RTC predictor of improved outcomes [68]. This potentially reflects the well-established role of self-efficacy as a treatment outcome predictor [3, 44] and the low levels of self-efficacy found in more severe PAU groups [21].

Despite such mixed findings and critiques, SoC/RTC has been defended on grounds of its clinical utility, whilst also paving the way for subsequent motivation orientated models in addictive behaviours [69]. However, it is argued that greater consideration and empirical testing of alternative theoretical frameworks of motivation are needed to advance the understanding of the motivational processes underlying changes in drinking [70]. *Self-determination theory* (SDT) [71] has been proposed as one candidate framework [72], particularly because of its substantial conceptual overlap with motivational interviewing [73, 74] and strong support in other domains of health behaviour change [75–78].

According to SDT, there are qualitatively distinct types of motivation that exist on a continuum of self-determination (see Table 2). Notably, the adoption of more self-determined motivation is described as *internalization* and aligns closely with our definition of problem recognition for this review (see Section. 2.). Internalization has been proposed to be necessary for maintaining healthy behaviour change over time [79], consistent with calls to develop problem recognition understanding [8]. Table 2 provides definitions and examples in relation to alcohol problem recognition for each of the five relevant regulatory styles described by SDT. While there is growing support for SDT in alcohol research [72], applications to motivation for change are limited. Given the conceptual overlap between alcohol problem recognition and motivation to change, theoretical advancements in motivation for change with empirical support will have important implications for understanding alcohol problem recognition.

**Table 2:** Definitions of Five Regulatory Styles on the Self-Determination Continuum of Motivation and Examples Related to Alcohol Problem Recognition

Regulatory Style	Regulatory Processes	Example
Amotivation	Nonintentional, Nonvaluing, Incompetence, Lack of Control	A person does not recognize their alcohol use as problematic and therefore does not intend to change
External Regulation	Compliance, External Rewards and Punishments	Others recognize a person’s alcohol use as problematic and pressure them to change
Introjected Regulation	Self-Control, Ego-Involvement, Internal Rewards and Punishments	A person makes changes to avoid guilt or to attain pride because of societal standards that indicate their drinking is problematic
Identified Regulation	Personal Importance, Conscious Valuing	A person makes changes because they recognize that their drinking is problematic and value the benefits of making a change
Integrated Regulation	Congruence, Awareness, Synthesis with Self	A person makes changes because they recognize that their drinking is problematic and have assimilated change with their other values and needs

*Note.* Content of the regulatory processes column was derived from [80; Fig. 1]

### Ambivalence

Closely aligned with motivation theories, *ambivalence* has been a widely used concept in addiction research and treatment, particularly via the use of motivationalinterviewing (MI) approaches [3, 81]. Ambivalence has been described as when a person perceives reasons for and against changing [82], and the “drinkers dilemma” to reflect dissonance arising from the co-occurrence of positive and negatives consequences of heavy drinking [83, 84]. As such, ambivalence may be seen to reflect some degree of problem recognition, albeit which is in tension with other conflicting beliefs about drinking. Notably, ambivalence is regarded as distinct from contemplation, denial, discrepancy, resistance or lack of motivation by MI experts [85].

In a notable study by Oser et al. (2010), an ‘ambivalence’ factor was generated by combining three measures including a single problem recognition item (*“To what extent do you feel that your drinking is a significant problem?”*), in combination with self-efficacy and depression^3^ measures. Each factor independently predicted treatment engagement and was associated with treatment outcomes, with the authors proposing further investigation into their novel formulation of ambivalence. Indeed, self-efficacy (or ‘confidence’ as per SoC/RTC measures) is an important variable in PAU outcomes [86, 87] and has been proposed as an important factor in problem recognition processes [8, 21].

Ambivalence, however, has been conflated with problem recognition. For example, a recent study finding associations between types of narcissism and various alcohol-related measures included ambivalence (as measured by the Readiness to Change Ruler for Decreased Drinking), but reported ambivalence as ‘problem recognition’ [88]. Such issues further point to the need for empirically derived conceptual work (e.g., as per Morris et al. 2021 [8]; see Fig. 1) to examine such concepts and their relationship to problem recognition.

### Treatment/help-seeking Intentions and Engagement

Whilst RTC and ambivalence measures are commonly used to assess help-seeking intentions or engagement with forms of treatment, aspects of help-seeking have been operationalized as an indicator of problem recognition. That is, a person is typically expected to recognise a problem if they are voluntarily seeking help for it. A systematic review of problem recognition amongst people with PAU (including hazardous use, as defined within the present review) by Smith et al. [10] included six studies where *perceived need for help*^4^ was used as a proxy for problem recognition. Studies were grouped to identify levels of problem recognition by type of measure. Within studies using perceived need for help as a proxy (n = 6) for problem recognition, 18% of participants were reported as having ‘problem recognition’. Within studies using explicit problem recognition measures (n = 7), 29% were classified as indicating problem recognition, with 59% of people within studies using RTC as a problem recognition proxy (n = 4). Such differences point to the need to develop not only conceptual clarification of problem recognition (see Fig. 1) and measures, but also the role of variables including PAU severity and treatment engagement (as discussed in 4.2).

### Expectancies

Alcohol *expectancies* are the extent to which a person associates positive or negative outcomes with drinking. Expectancies may form an important relationship with problem recognition such that increasing problem recognition would be expected to predict/reflect re-appraisals of positive and negative alcohol expectancies. In a systematic review of alcohol treatment outcome predictors, expectancies were identified as one of the strongest predictors, alongside alcohol-related self-efficacy, motivation to change and treatment goal [44]. Expectancies are likely to encompass other important self-evaluative appraisals associated with problem recognition including identity shifts [34].

### Normative Misperception

*Normative misperception* reflects the extent to which people inaccurately assess the behaviour of others (e.g., the proportion of people who drink above guideline levels), with important implications according to *social norms theory* [89]. As such, normative misperception indicates low problem recognition as a result of over-estimating the drinking of others. Indeed, large degrees of normative misperception has been identified in PAU groups based on a discrepancy between AUDIT scores and evaluations of other’s alcohol use. For instance, Garnett et al. [46] found in a general population sample (n = 9,820) that 25.4% of people with probable alcohol dependence and 36.6% of harmful alcohol users evaluated their alcohol use to be average or less than average, whilst non-PAU groups (i.e., lower risk drinkers) more accurately gauged their own alcohol use relative to others.

### Unrealistic Optimism and Susceptibility Biases

Similarly to normative misperception, *unrealistic optimism* (or *optimism bias*) reflects a cognitive bias in which people underestimate their own susceptibility to negative health outcomes relative to others [90, 91]. As such, optimism biases indicate low problem recognition as a result of cognitive biases which are likely rooted in self-protective cognitive processes [21], In a recent study, a positive association was found between unrealistic optimism and level of alcohol use (*r* = 0.50), such that the higher the PAU severity the higher the level of unrealistic optimism [21]. Similar associations for higher PAU severity were found with self-rated threat (*“How serious are the health consequences of regularly exceeding the recommended drinking guidelines of 14 units per week?”*) and susceptibility (*‘My chances of experiencing alcohol-related health problems such as liver damage or some cancers in the future if I regularly drink above the recommended drinking guidelines are…’*). As such, the authors propose optimism biases and implicit defensive processing of alcohol risk information are strategies to maintain low problem recognition in PAU groups, as proposed by Morris and colleagues (2021; see Fig. 1). These personal evaluations of risk are proposed as closely aligned to the proposed operationalization of problem recognition within the current review, albeit that the above examples only extend to health risks, and therefore do not account for a range of other ‘problems’ associated with alcohol use.

### Qualitative Insights into Problem Recognition

To our knowledge, no qualitative work has been undertaken that specifically seeks to identify and conceptualise problem recognition amongst PAU groups. However, a substantial body of literature captures how PAU groups identify and construct alcohol-related problems and associated discourse, indicating how many PAU groups reject their drinking behaviours as ‘problematic’, thus indicative of low problem recognition [52–54, 92–95].

An important factor relating to problem recognition is therefore how alcohol problems are perceived and how this relates to one’s own use. For instance, people with PAU engaging in treatment or ‘recovery’ contexts exhibit higher levels of problem recognition [11, 96]. As such, a consistent theme in recovery accounts has been identified as ‘being honest with oneself’ [6, 97, 98], whilst a requirement of Alcoholics Anonymous participation is self-identification as an “alcoholic” [99]. Nonetheless, labelling theories and qualitative accounts highlight the complex self-evaluations that come with treatment engagement [31, 100, 101], thus highlighting how problem recognition may fluctuate during ‘recovery’ [102].

In lay terms, ‘denial’ is commonly applied to describe a ‘failure’ to acknowledge a perceived alcohol problem [103], but its use presents a number of important issues including its stigmatizing connotations and failure to capture the more complex nature of problem recognition processes [8, 23]. For lower severity PAU groups (i.e., hazardous or harmful patterns not typically meeting AUD criteria), many qualitative accounts have identified how low problem recognition is maintained via discursive practices that create a ‘responsible’ non-problematic drinking identity [104]. This practice draws on a dichotomized dominant master narrative around public perceptions of alcohol problems orientated around an alcoholism model which frames alcohol problems as a severe, individually located and pathological condition [26]. In turn, many PAU groups engage in *othering* by drawing on severe stereotypes, in turn driving stigma towards those perceived as ‘problem drinkers’ [24, 104]. This literature has been proposed to be consistent with empirical findings about how low problem recognition is maintained in PAU via defensive processing, unrealistic optimism [21] and othering processes [31, 104]. However, qualitative work to specifically examine problem recognition processes in accordance with proposed conceptual models would be valuable.

### Discussion, Limitations, and Future Directions

This review has taken a non-systematic narrative approach to author identified literature on PAU problem recognition concepts and measurement. A large literature on PAU and other addiction behaviour changes processes exists, thus we have only briefly covered key relevant concepts and measures. Nonetheless, we have identified direct and proxy measures for PAU problem recognition based on a broad definition of PAU which spans hazardous and harmful use in addition to AUD. We propose problem recognition is important to measure as a continuous variable (e.g., as per the SPRS scale: see 3.1) given statistical advantages such as sensitivity [105] and the non-dichotomous nature of alcohol use and problems [21]. However, categorical measures have still been effectively utilized to identify the significance of problem recognition in alcohol-related outcomes [e.g., 10, 12].

We propose further conceptual development of problem recognition is needed alongside theoretically informed testing of problem recognition measures and other key variables in PAU behaviour change. A conceptual model incorporating the multi-faceted nature of problem recognition processes amongst harmful drinkers^5^ (see Fig. 1) has been proposed as a basis for further empirical testing [8]. Within this model, PAU problem recognition has been proposed as a complex and dynamic process reflecting multiple potential cues (i.e., exposure to personally relevant information about the risk/harms), moderating or mediating factors (e.g., beliefs about the nature of PAU, perceptions of stigma, self-efficacy etc.) and potential outcomes (e.g., self-change, help-seeking). However, although defined as an explicit evaluation, it has been proposed that PAU problem recognition must also account for important implicit processes which likely contribute to many of the concepts identified in Section. 4, particularly ‘defensive processing’ of relevant but threatening information [21].

One key issue for future development is to consider the suitability of problem recognition concepts and measures whilst considering the broad scope of alcohol use and problems and related limitations to PAU concepts [26], including AUD criteria which excludes many groups at risk of or experiencing harm from their alcohol use [1, 106]. In the context of substance use disorder (SUD) [37, 107] and mental health [108], *insight* has been operationalized to reflect problem recognition processes, but typically aligned within ‘illness’ models which have been argued to hinder problem recognition in non-help seeking PAU groups [26]. However, problem recognition may not be appropriate or useful to explore within certain clinical populations with alcohol-related brain damage/significant cognitive impairment, and thus the application of ‘insight’ (or ‘anosognosia’^6^) as a distinct approach may be more valid in such contexts [50]. As such, in addition to the complex and heterogeneous nature of alcohol use and associated harms, a clear consideration for the suitability of problem recognition concepts is PAU severity and diagnostic thresholds [109]. Indeed, existing literature highlights how PAU problem recognition (or indicative measures) varies significantly by severity [9, 21, 29, 35, 40, 46]. This includes implications for how lower severity ‘problems’ (notably hazardous consumption or sub-clinical problems^7^) may legitimately be argued as ‘non-problematic’ since there may yet be an absence of clear *harm* (rather than risk).

This highlights issues inherent with how PAU and relevant thresholds are determined, and in turn, whether ‘problem’ recognition requires differential approaches and/or ontologies for different levels of alcohol use/risk and/or problems. Accordingly, it may be argued that *risk recognition* is more appropriate at hazardous consumption levels, but creating separate scales/concepts raises similar problems to those well established in creating AUD thresholds [110, 111]. A further consideration is that the term ‘problem’ is itself normative and potentially stigmatizing via labelling someone as a ‘problem drinker’. Evaluating problem recognition status following drinking reductions or abstinence also requires careful consideration given variations in interpretation of ‘recovery’ and what constitutes PAU [2]. These issues also reflect the strong social and identity processes that are inherent within how alcohol use and problems are conceptualized across cultures [17, 18, 26], in turn, directly influencing how ‘alcohol problems’ are perceived, assessed and operationalized across public and professional domains. Indeed identity processes have been shown to directly influence problem recognition, including through identity threat (i.e., self-stigma) [31] and drinker identity processes [36, 112]. As such, the cultural and social processes involved in alcohol use and evaluation are also important to consider in order to avoid excessive focus on individual processes in addressing PAU [26].

## Conclusion

Problem recognition appears to be a crucial factor in PAU behaviour change. Despite this, there has been limited conceptual approaches to defining and testing PAU problem recognition. Related concepts, particularly focusing on ambivalence and motivation for change, are also lacking coherent theoretical and empirical development and often conflated with problem recognition. Developing understanding of problem recognition, its key antecedents and its mediating role in PAU outcomes across differing levels of PAU severity offers important opportunities to advance the effectiveness of efforts to reduce PAU.

## Data Availability

No datasets were generated or analysed during the current study.

## Footnotes

1 We use the term problematic alcohol use (PAU) in view of the importance of capturing the wide spectrum of alcohol problems and risk, as per the proposed problem recognition working definition (see Sect. 2.). We also use the term alcohol use disorder (AUD) when specifically referring to PAU which meets AUD criteria (e.g., when operationalized as AUD as per DSM-5 [113] within a specific study).

2 We acknowledge that ‘alcoholic’ terminology may be important in self-labelling contexts, but has been found to reduce problem recognition in other contexts [31].

3 A depression measure was included as the authors propose depression often stems from a discrepancy between one’s behaviours and values, and is therefore a key facet of ambivalence [114]

4 Perceived need for help included studies where help was sought or self-reported as needed but not accessed. Help included formal or informal sources.

5 A defined AUD category based on consumption thresholds at which harms are present (i.e., drinking above ‘hazardous’ levels).

6 ‘Anosognosia’ has been used in neuropsychiatric contexts to describe the impairments in awareness of addiction problems linked to cognitive impairment [e.g., 105]. It’s etymology stems from its Greek meaning “without knowledge of disease”.

7 Thus typically not qualifying as ‘AUD’ based on many definitions/threshold approaches.
